# GPVI and GPIbα Mediate Staphylococcal Superantigen-Like Protein 5 (SSL5) Induced Platelet Activation and Direct toward Glycans as Potential Inhibitors

**DOI:** 10.1371/journal.pone.0019190

**Published:** 2011-04-28

**Authors:** Houyuan Hu, Paul C. J. Armstrong, Elie Khalil, Yung-Chih Chen, Andreas Straub, Min Li, Juliana Soosairajah, Christoph E. Hagemeyer, Nicole Bassler, Dexing Huang, Ingo Ahrens, Guy Krippner, Elizabeth Gardiner, Karlheinz Peter

**Affiliations:** 1 Baker IDI Heart & Diabetes Institute, Melbourne, Victoria, Australia; 2 Department of Cardiology, Southwest Hospital, Third Military Medical University, Chongqing, China; 3 Department of Medicine, Monash University, Melbourne, Victoria, Australia; 4 Australian Centre for Blood Diseases, Monash University, Melbourne, Victoria, Australia; The University of Hong Kong, Hong Kong

## Abstract

**Background:**

*Staphylococcus aureus (S. aureus)* is a common pathogen capable of causing life-threatening infections. Staphylococcal superantigen-like protein 5 (SSL5) has recently been shown to bind to platelet glycoproteins and induce platelet activation. This study investigates further the interaction between SSL5 and platelet glycoproteins. Moreover, using a glycan discovery approach, we aim to identify potential glycans to therapeutically target this interaction and prevent SSL5-induced effects.

**Methodology/Principal Findings:**

In addition to platelet activation experiments, flow cytometry, immunoprecipitation, surface plasmon resonance and a glycan binding array, were used to identify specific SSL5 binding regions and mediators. We independently confirm SSL5 to interact with platelets *via* GPIbα and identify the sulphated-tyrosine residues as an important region for SSL5 binding. We also identify the novel direct interaction between SSL5 and the platelet collagen receptor GPVI. Together, these receptors offer one mechanistic explanation for the unique functional influences SSL5 exerts on platelets. A role for specific families of platelet glycans in mediating SSL5-platelet interactions was also discovered and used to identify and demonstrate effectiveness of potential glycan based inhibitors *in vitro*.

**Conclusions/Significance:**

These findings further elucidate the functional interactions between SSL5 and platelets, including the novel finding of a role for the GPVI receptor. We demonstrate efficacy of possible glycan-based approaches to inhibit the SSL5-induced platelet activation. Our data warrant further work to prove SSL5-platelet effects *in vivo*.

## Introduction

*Staphylococcus aureus* (*S. aureus*) is one of the most common and dangerous bacterial pathogens to humans. It can cause a range of diseases including life-threatening thromboembolic diseases such as infective endocarditis [1], [2] and disseminated intravascular coagulation (DIC) [3]. *S. aureus* is increasingly found in the community and hospitals, and is one of the most common bacterium isolated from blood cultures. Additionally, its growing resistance to multiple drugs means this pathogen presents a major clinical challenge [4].

The interaction of *S. aureus,* via a variety of surface-associated proteins with platelets has recently been recognized to play a significant pathophysiological role in *S. aureus*-associated diseases [5]. These direct interactions between *S. aureus* and platelets result in bacteria-platelet aggregates which, for example, are characteristic of *S. aureus* endocarditis [6]. *S. aureus* is known to secrete the Staphylococcal superantigen-like proteins (SSLs) that are structurally homologous to the superantigens (SAg), but do not seem to exhibit the same functions [7]. The SSLs are a group of related genes which are all clustered on a genomic island [8], [9], [10]. This pathogenicity island contains between 7 and 11 SSL genes [9], [11] and is present in all strains of *S. aureus* examined to date [11].

While SSLs have previously been implicated in *S. aureus* virulence [12], recent studies showed that two of these toxins, SSL5 and SSL11, can bind to P-selectin glycoprotein ligand-1 (PSGL-1) on granulocytes to inhibit P-selectin-mediated neutrophil rolling and the subsequent migration of neutrophils to sites of infection [7], [13], [14], and inhibit leukocyte activation by chemokines and anaphylatoxins [15]. PSGL-1 is structurally and functionally related to the GPIbα subunit of the platelet GPIb-IX-V receptor complex. Indeed, SSL5 has recently been demonstrated to cause platelet activation, associated with interactions between SSL5 and either GPIbα or GPIIb/IIIa [16]. Furthermore, as these receptors are membrane-associated sialomucins containing large clusters of O-linked sugar chains and have been shown to bind P-selectin [17], [18], [19], [20], a role for glycans has been implicated [16].

In this study, we independently corroborate that SSL5 can induce *in vitro* platelet activation and also possesses the ability of binding to platelet membrane receptor GPIbα, with the sulphated-tyrosine residues playing a significant role. We also identify the novel interaction between SSL5 and the collagen receptor GPVI [21]. Furthermore, our study defines specific glycan families which are important in mediating the SSL5-platelet interaction, and demonstrate potential glycan based therapeutic approaches to inhibit SSL5-induced platelet activation.

## Results

### SSL5 specifically binds to human platelets in a concentration-dependent manner

**Figure 1:**
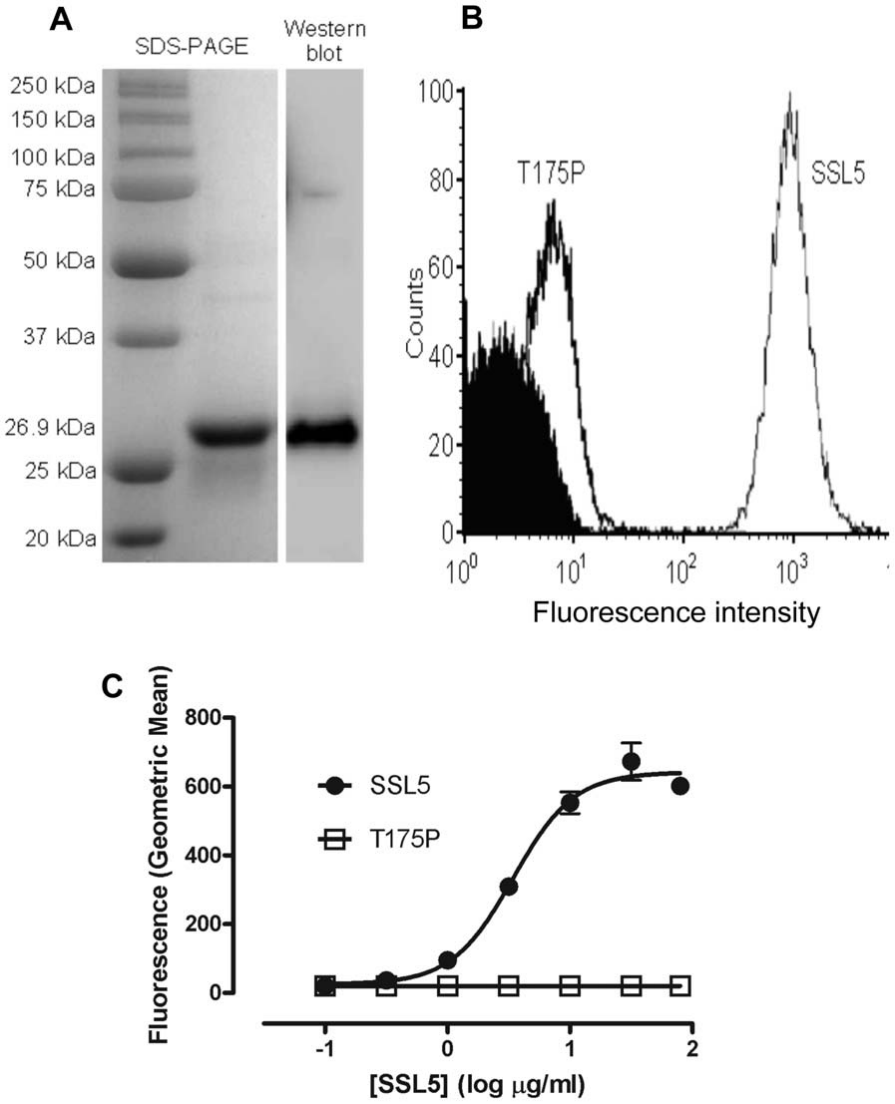
Analysis of purified recombinant SSL5 and flow cytometric examination of SSL5 binding to human platelets. (**A**) Purification of a ∼27 kDa SSL5 protein from BL21 *E. coli*. Samples were analyzed by SDS-PAGE and Coomassie blue staining and by Western blot using anti-His6 mAb as described in Supplemental Materials and Methods S1. (**B**) A representative flow cytometry histogram of platelets incubated with 10 µg/ml SSL5 or SSL5 mutant T175P. (**C**) Washed platelets were incubated with 0.1–80 µg/ml of either SSL5 or T175P SSL5 followed by Alexa Fluor 488-conjugated anti-penta-His mAb and fluorescence intensity was measured by flow cytometry. Data is expressed as geometric mean fluorescence intensity ± SEM of three independent experiments.

Figure 1A shows purified recombinant SSL5 migrating as a single band of ∼27 kDa. Purified SSL5 has been previously shown to bind to the human leukemic HL60 monocytic cell line [13], [22] and we confirmed that our recombinant SSL5 protein, but not a non-functional mutant form of SSL5 carrying a T175P point mutation (T175P) [22] binds to HL60 cells by flow cytometry. In addition, SSL5 blocked the binding of anti-PSGL-1 mAb KPL-1 to HL60 cells (data not shown). SSL5 binding to human washed platelets, detected by flow cytometry, increased in a concentration-dependent manner, contrasting with T175P which did not (figures 1B and 1C).

### Activation and inhibition of platelets by SSL5

**Figure 2:**
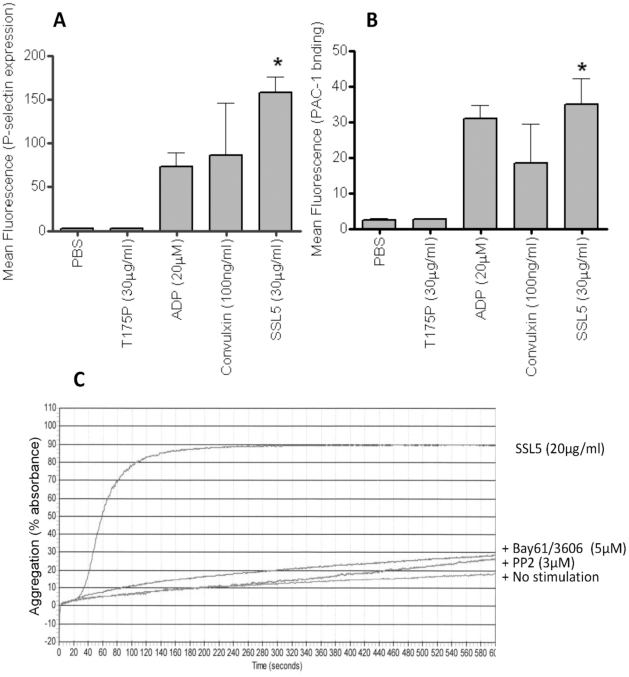
SSL5 activates human platelets and induces syk and src dependent platelet aggregation. (**A**) Levels of anti-P-selectin mAb or (**B**) PAC-1 binding were measured by flow cytometry in samples of washed platelets after incubation with PBS, 20 µM ADP, 100 ng/ml convulxin, or 30 µg/ml SSL5 or T175P SSL5. (**C**) Representative aggregation trace obtained in washed platelets, induced by 20 µg/ml SSL5 or no stimulation (PBS). Aggregation was strongly inhibited by pre-treatment of platelet with either a syk kinase inhibitor (BAY61-3606, 5 µM), or a src inhibitor (PP2, 3 µM). Images are representative of three independent experiments. * *P*<0.05.

**Figure 3:**
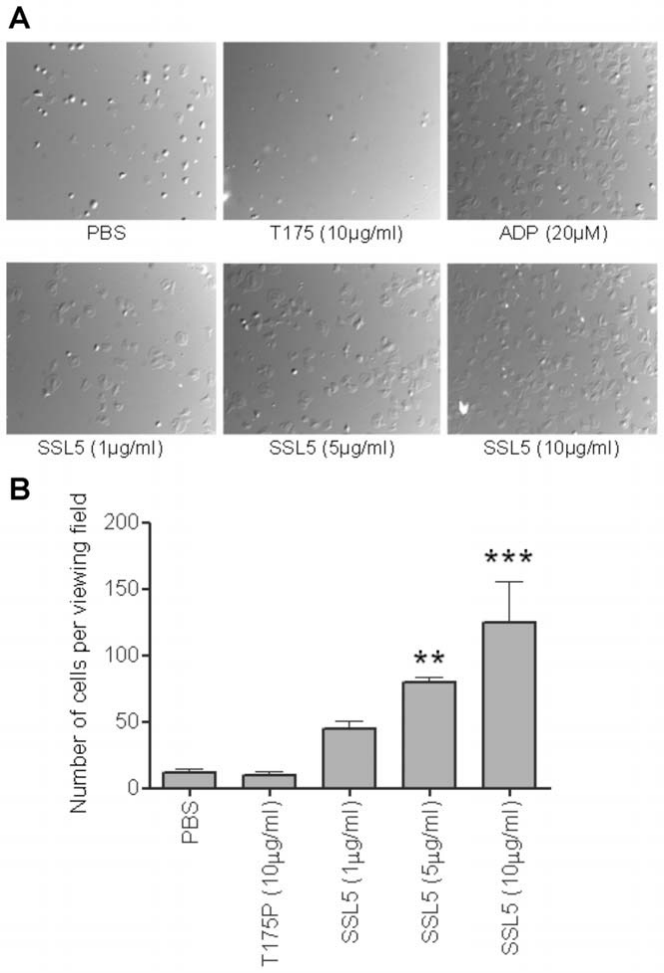
SSL5 induces spreading of platelets on fibrinogen. Gel filtered platelets (GFP) were incubated with 20 µM ADP, 1–10 µg/ml SSL5, 10 µg/ml T175P (mutant SSL5) at 37°C for 30 minutes on fibrinogen. (**A**) Representative pictures were obtained by DIC (×60) following adhesion. (**B**) Presented data are means ± SEM of four independent experiments using five separate fields per experiment. ** *P*<0.01, *** *P*<0.001 compared to control (PBS).

Surface levels of P-selectin (figure 2A), and the activation of the integrin GPIIb/IIIa, using the active conformation-specific mAb PAC-1 (figure 2B) were assessed as measures of platelet activation. Treatment with SSL5 but not T175P induced increased levels of P-selectin and active GPIIb/IIIa on platelets, to levels comparable to those observed after P2Y- or GPVI-dependent activation of platelets. Aggregation of human washed platelets after treatment with SSL5, but not the mutant SSL5 (data not shown), was confirmed by light transmission aggregometry (figure 2C), and which was inhibited by prior incubation (5 min) with either a spleen tyrosine kinase (BAY61-3606, 5 µM) [23] or src (PP2, 3 µM) [24] inhibitor. SSL5-treated platelets also firmly adhered and spread on a fibrinogen-matrix under static conditions in a dose-dependent manner (figure 3A and 3B).

### Identification of SSL5-binding epitopes on GPIbα

**Figure 4:**
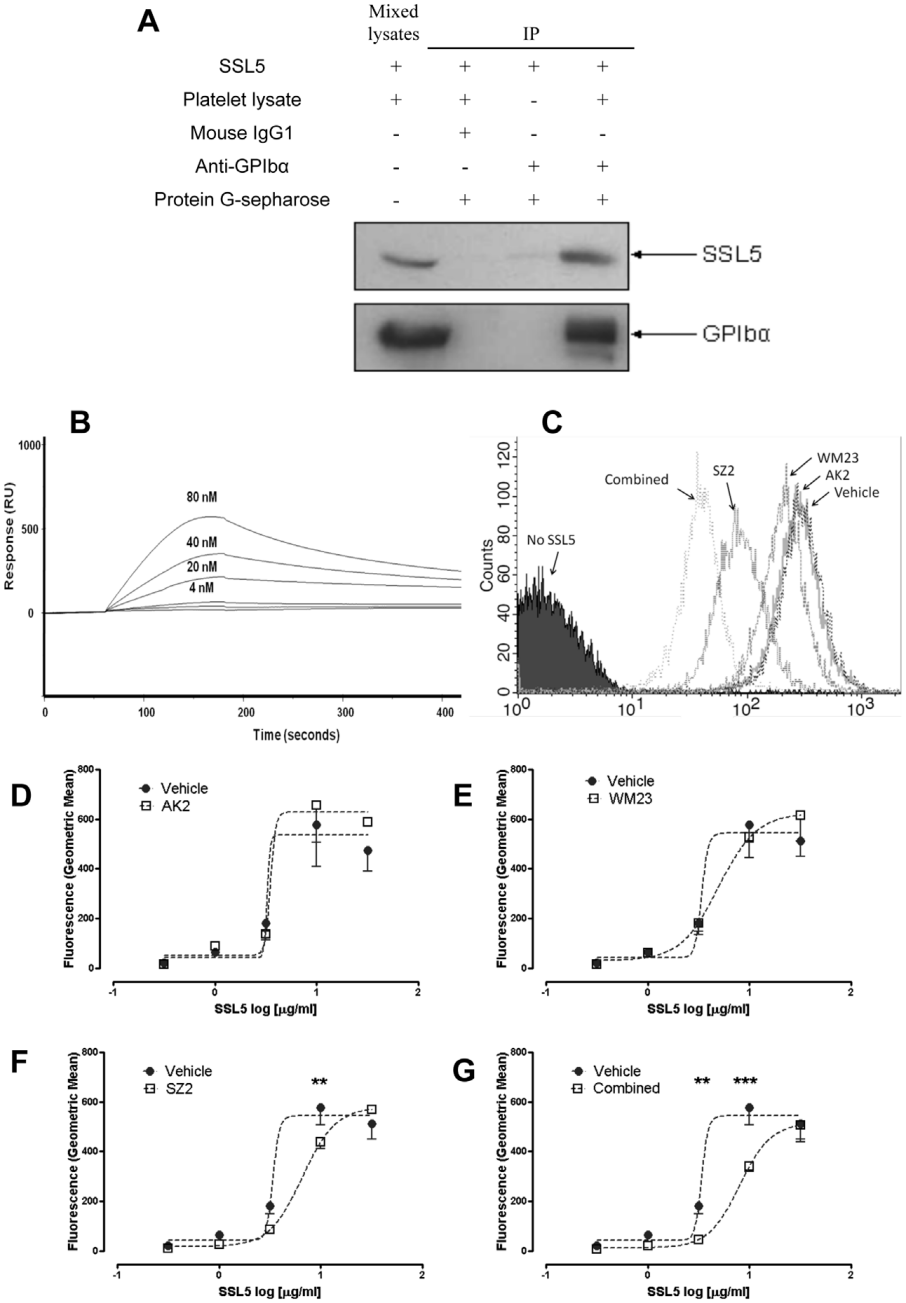
Sulfated-tyrosine residues of GPIbα constitute an important binding region for SSL5. (**A**) Purified SSL5 proteins incubated with human washed platelet lysates were co-immuno-precipitated by an anti-GPIbα mouse mAb (WM23) and subjected to immunoblotting probed with an HRP-conjugated anti-His antibody (top panel). The filter was re-probed with mouse anti-human GPIbα mAb to show GPIbα was present in the immunoprecipitate (bottom panel). Negative controls were mouse IgG1 or without platelet lysates. Directly mixed platelet lysates with SSL5 used as positive control. (**B**) Recombinant, affinity-purified Fc fusion proteins of GPIbα were immobilized by injection over CM5-streptavidin biosensor chips and the binding response of SSL5 with GPIbα was determined. (**C**) Representative histogram of SSL5-platelet binding at 3 µg/ml in presence of vehicle or anti-GPIbα antibodies. Washed platelets incubated with vehicle alone or 3 µg/ml of GPIbα antibodies (**D**) AK2 and (**E**) WM23 and (**F**) SZ2, individually, or (**G**) in combination for 15 minutes at 37°C prior to addition of 0.3–30 µg/ml SSL5 and binding determined by flow cytometry. SZ2, but not AK2 or WM23, significantly reduced binding, whilst use of all three antibodies in combination further reduced the amount of SSL5 binding. Data are presented as mean ± SEM (** *P*<0.01, *** P<0.001 by paired two-way ANOVA).

Using immunoprecipitation we confirmed the co-isolation of SSL5 with PSGL-1 from HL60 cells using an anti-PSGL-1 mAb (data not shown), consistent with previous reports of SSL5 characteristics [13], [22]. Using anti-GPIbα (WM23) or isotype control (mouse IgG1), SSL5 was detected in GPIb-specific immunoprecipitates but not isotype controls (figure 4A), confirming a molecular interaction between GPIbα and SSL5. Direct interaction between SSL5 and GPIbα was confirmed by surface plasmon resonance using recombinant fusion proteins containing the N-terminal ligand-binding region fused to a C-terminal human IgG domain (figure 4B). No interaction was seen when a control human IgG domain alone was used or when the SSL5 mutant T175P was used as the analyte (data not shown). Concentrations of SSL5 above 80 nM were also assessed but found to display inconsistent binding characteristics under the experimental conditions used; possibly due to the cationic nature of the molecule.

Identification of binding sites on GPIbα was done by pre-incubating platelets with mAbs raised against separate epitopes of GPIbα, and the subsequent binding of SSL5 assessed using flow cytometry. Binding of SSL5 (3 and 10 µg/ml) and platelets, was significantly inhibited by the anti-GPIbα mAb SZ2 (p<0.01), identifying the sulphated-tyrosine region as a key binding site. The mAbs AK2 (first leucine-rich repeat region) and WM23 (macroglycopeptide region) only weakly inhibited this interaction, whereas the greatest level of inhibition was achieved by co-incubating all three mAbs with platelets (p<0.001; figure 4C–G).

### Interaction between SSL5 and GPVI

**Figure 5:**
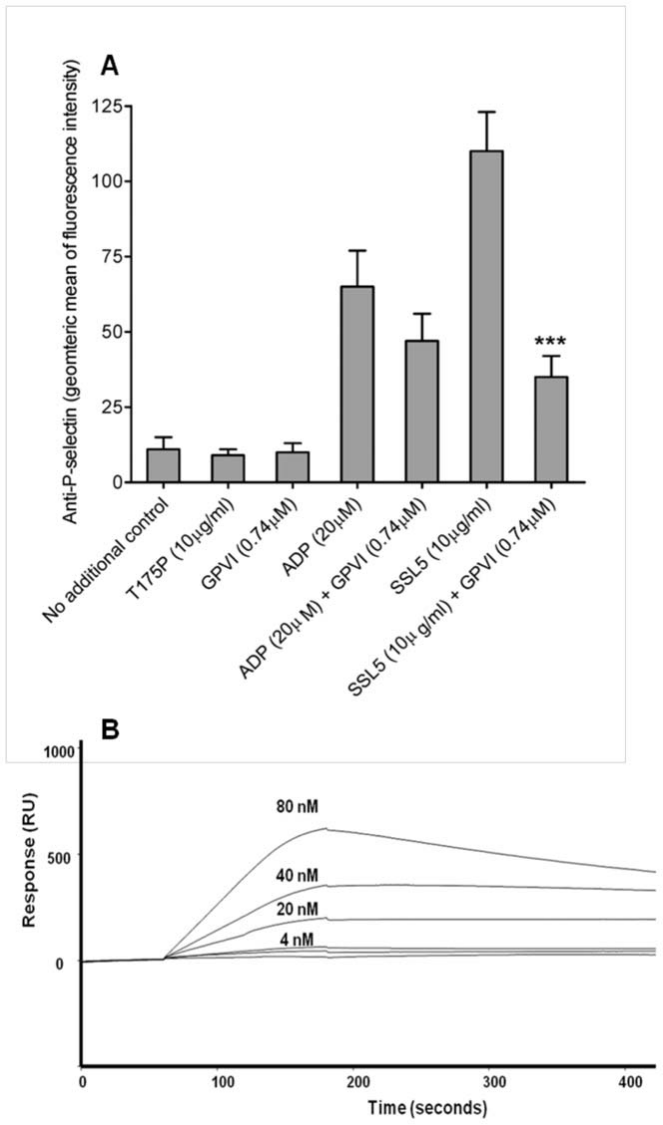
Platelet GPVI receptor was identified as a novel binding partner for SSL5. (**A**) Flow cytometry was used to assess the inhibition of P-selectin expression on human platelets following pre-incubation of SSL5 with recombinant GPVI. Bar graphs represent the geometric mean fluorescence intensity (means ± SEM) across six individuals (n = 6). ***p<0.001 *vs* SSL5 only. (**B**) Direct interaction was confirmed by surface plasmon resonance using GPVI-Fc chimera.

Preincubation of SSL5 with a recombinant ectodomain fragment of GPVI, containing the two immunoglobulin-like domains with the ligand binding region but not the mucin-like domain, equivalent to twice the concentration of SSL5 inhibited SSL5-induced, but not ADP-induced, P-selectin expression by 75% (p<0.001; figure 5A). A direct interaction was demonstrated using surface plasmon resonance using a GPVI recombinant protein consisting of N-terminal ligand-binding region (residues 21–234) fused to a C-terminal human IgG domain (figure 5B). No interaction was found between GPVI or human IgG domain coating when the T175P analyte was used (data not shown). Concentrations of SSL5 above 80nM were also assessed but found to display inconsistent binding characteristics under the experimental conditions used; possibly due to the cationic nature of the molecule.

### SSL5 glycan binding specificity and ability to inhibit SSL5 effects *in vitro*

**Figure 6:**
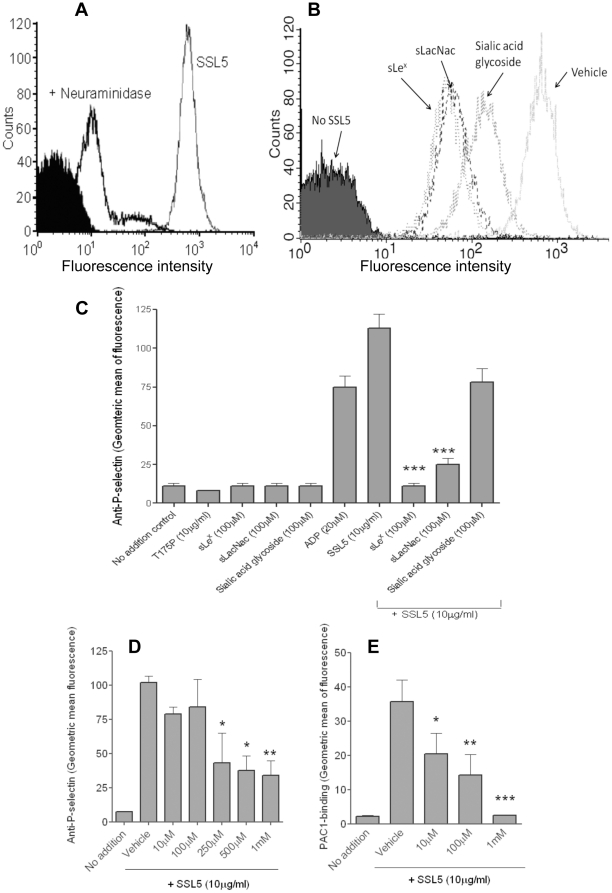
Inhibition of SSL5-induced platelet activation by sLe^X^, sLacNac, sialic acid glycoside and bimosiamose. (**A**) Binding of SSL5 to untreated or neuraminidase-treated platelets; Filled histogram: anti-His-tag antibody alone. (**B**) Binding of SSL5 in presence of vehicle or 100 µM sLe^X^, sLacNac, sialic acid glycoside. (**C**) Flow cytometry to assess the ability of a range of glycans to inhibit SSL5-induced expression of P-selectin. (**D, E**) Bimosiamose inhibited SSL5-induced platelet activation, both anti-P-selectin and PAC-1 binding. Bar graph data are presented as mean ± SEM (n = 4-6). *p<0.05, *** *p*<0.01 *vs* SSL5 or vehicle.

Binding of SSL5 to platelets treated with neuraminidase, to remove sialic acid from the platelet surface, was almost completely lost (figure 6A), indicating a role for sialic acid residues in platelet-SSL5 interactions. A glycan microarray was undertaken and screened 377 different glycans for binding ability. The strongest 20 binders are presented in Table 1. Notably, the trisaccharide sialyllactosamine (sLacNac - Neu5Acα2-3Galβ1-4GlcNAc) terminus was present in all but one (entry 12) of the high affinity glycans, and the PSGL-1 tetrasaccharide sialyl Lewis X (sLe^X^: Neu5Acα2-3Galβ1-4(Fucα1-3)GlcNAc) was well represented in the glycans with highest affinity. Additionally, SSL5 was found to bind to eight of the ten strongest SSL11-binding glycans [14]. Using recombinant SSL5 we investigated the use of glycans as potential drug candidates for SSL5 blocking properties. Flow cytometric analysis showed that incubation of each of the glycans sLe^X^, sLacNac, sialic acid glycoside, at a final concentration of 100 µM both reduced the levels of SSL5 binding (figure 6B) and inhibited the platelet-activating effect of SSL5. The sLacNac and sialic acid glycoside glycans were able to inhibit SSL5 (10 µg/ml)-induced platelet activation by 86% (p<0.001) and 35% (p = 0.059), respectively (figure 6C). No effect was observed on ADP-induced activation (data not shown). The glycan-based drug candidate bimosiamose, inhibited both SSL5-induced P-selectin expression and GPIIb/IIIa activation in a concentration-dependent manner between the concentrations of 10 µM and 1 mM (figures 6D and 6E).

**Table 1:** SSL5 binding glycans as determined by a glycomic array containing 377 different glycans.

Entry	Glycan Name	Mean RFU
1^1^	Neu5Acα2-3Galβ1-4(Fucα1-3)GlcNAcβ1-3Galβ-Sp8	17612
2^1^	Neu5Acα2-3Galβ1-4(Fucα1-3)GlcNAcβ1-3Galβ1-4GlcNAcβ-Sp8	15918
3	Neu5Acα2-3Galβ1-4GlcNAcβ1-3Galβ1-4GlcNAcβ1-3Galβ1-4GlcNAcβ–Sp0	15827
4^1^	Neu5Acα2-3Galβ1-4(Fucα1-3)GlcNAcβ–Sp8 ***(PSGL-1)***	15098
5	Neu5Acα2-3Galβ1-4GlcNAcβ1-3Galβ1-4GlcNAcβ-Sp0	14449
6^1^	Neu5Acα2-3Galβ1-4(Fucα1-3)GlcNAcβ–Sp0 ***(sLe^X^)***	14264
7^1^	Neu5Acα2-3Galβ1-4(Fucα1-3)GlcNAcβ1-3Galβ1-4(Fucα1-3)GlcNAcβ1-3Galβ1-4(Fucα1-3)GlcNAcβ–Sp0	13772
8	Neu5Acα2-3Galβ1-4GlcNAcβ–Sp8 ***(sLacNac)***	13546
9	Neu5Acα2-3Galβ1-4GlcNAcβ1-2Manα1-3(Neu5Acα2-3Galβ1-4GlcNAcβ1-2Manα1-6)Manβ1-4GlcNAcβ1-4GlcNAcβ-Sp12	12517
10	Neu5Acα2-3Galβ1-4GlcNAcβ1-3Galβ1-3GlcNAcβ-Sp0	10202
11	Neu5Acα2-3Galβ1-4GlcNAcβ–Sp0	9762
12	Neu5Acα2-3GalNAcβ1-4GlcNAcβ-Sp0	9449
13	Neu5Acα2-6Galβ1-4GlcNAcβ1-2Manα1-3(Neu5Acα2-3Galβ1-4GlcNAcβ1-2Manα1-6)Manβ1-4GlcNAcβ1-4GlcNAcβ-Sp12	9385
14	Neu5Acα2-3Galβ1-3(Neu5Acα2-3Galβ1-4GlcNAcβ1-6)GalNAc–Sp14	9183
15	Galβ1-3-(Neu5Acα2-3Galβ1-4GlcNAcβ1-6)GalNAc-Sp14	8578
16^1^	Neu5Gcα2-3Galβ1-4(Fucα1-3)GlcNAcβ-Sp0	8453
17	Neu5Acα2-3Galβ1-4GlcNAcβ1-2Manα1-3(Neu5Acα2-6Galβ1-4GlcNAcβ1-2Manα1-6)Manβ1-4GlcNAcβ1-4GlcNAcβ-Sp12	8043
18	Neu5Acα2-3Galβ1-4GlcNAcβ1-3GalNAc-Sp14	7924
19	Neu5Acα2-3Galβ1-3(Neu5Acα2-3Galβ1-4)GlcNAcβ-Sp8	7865
20^1^	(Neu5Acα2-3-Galβ1-3)(((Neu5Acα2-3-Galβ1-4(Fucα1-3))GlcNAcβ1-6)GalNAc–Sp14	7498

1 Glycan contains sLe^x^ tetrasaccharide (which includes sLacNac trisaccharide).

A ‘hit’ was defined as any glycan wherein the binding signal from fluorescent-labelled SSL5 (200 µg/ml) exceeded two standard deviations more than 10% of the strongest binding glycan. The best 20 of the 377 hits are shown. Sp0 (ethoxyamine), Sp8 (propyloxyamine), Sp12 (aspartamide) and Sp14 (threonine) represent glycosidic linkers used to attach the glycan to the microarray surface. RFU  =  Relative Fluorescence Units.

## Discussion

It has recently been shown that SSL5, an exotoxin secreted by *S. aureus*, can interact with the platelet glycoproteins GPIbα causing platelet activation [16]. Using flow cytometry, immunoprecipitation, a glycan binding array and surface plasmon resonance, we have independently confirmed GPIbα as a receptor for SSL5 on platelets and identified some of the molecular regions of GPIbα that support SSL5 binding to the platelet surface. Furthermore, our data identifies GPVI as also potentially contributing to the localization of SSL5 with the platelet surface.

The recent publication by de Haas *et al* [16], did not specify the precise SSL5-binding region on GPIbα, although the authors reported that the AK2 antibody, that binds to an epitope within the first leucine-rich-repeat of GPIbα, had little effect on SSL5 binding. In this study, we used a range of epitope-specific GPIbα antibodies to examine the nature of SSL5/GPIbα binding. We found that the SZ2 antibody, which binds to the anionic sulphated-tyrosine region, had a significant inhibitory effect on SSL5 binding to platelets. When the GPIbα-specific AK2, WM23 and SZ2 antibodies were used in combination, more than 75% inhibition of SSL5 binding was observed. We report here a PSGL-1-mediated binding of SSL5 to HL60 cells, and other members of the SSL family have been reported to bind PSGL-1 which contains an N-terminal sulfated-tyrosine sequence remarkably similar to the anionic region of GPIbα. P-selectin utilises these negatively charged regions to bind to both PSGL-1 and GPIbα. GPIbα is a sialomucin [25] and can bind ligands via sLe^x^-related carbohydrates. Our finding that the SSL5 mutant T175P, which could no longer bind to glycans, also lost the ability to bind to GPIbα, is consistent with glycans contributing the main component of SSL5 binding to GPIbα. Whilst antibody steric hindrance [26] or the preferential interaction of SSL5 with carbohydrate moieties within GPIbα antibodies cannot be formally excluded as an explanation of our observations using antibodies with different binding epitopes on GPIbα, other potential binding determinants within GPIbα may be involved; for example it is conceivable that the mucin-rich region within the macroglycopeptide region of GPIbα may modulate the binding of SSL5. Further work is required to examine this point.

Our identification of the collagen receptor GPVI as another binding site for SSL5 provides, along with GPIbα, a mechanistic basis for understanding SSL5 induced platelet activation, as GPVI is known to couple with the robust collagen-induced signalling pathway [21]. The inhibition of SSL5-induced platelet aggregation by the syk kinase inhibitor, Bay61-3606 and the src inhibitor PP2, supports this hypothesis. Furthermore, it has previously been shown that these two glycoproteins and their respective signalling pathways can directly interact. A functional complex between GPIbα and GPVI on platelets has been reported, and an important role for the sulphated-tyrosine region GPIbα was identified [27]. It is possible that both platelet receptors may contribute SSL5 binding determinants, consistent with surface plasmon resonance data and inhibition of SSL5 binding and activation of platelets by soluble recombinant GPVI.

As both of the glycoproteins identified are characterised by *N-* and *O*-linked carbohydrate-rich extracellular regions [28], [29], we sought to identify specific glycans that have high binding affinity for SSL5. A glycan array undertaken at Core H of the Consortium for Functional Glycomics revealed a specificity of SSL5 for glycans containing the sLacNac terminus. Interestingly, a similar specificity has been previously reported for SSL11 [14]. Found on GPIbα [30], the sLacNac trisaccharide was present in nineteen of the twenty SSL5-binding glycans that demonstrated the highest affinity in binding.

The binding of SSL5 to platelet GPIbα and GPVI, subsequent induction of platelet activation, and inhibition of specific adhesion abilities, may represent a pathogenic mechanism of *S. aureus* infection. Whilst, further work is required to determine the effects of this mediator *in vivo*, the effects in experiments performed *in vitro* suggest SSL5 is a unique potential therapeutic target. To this end, we investigated the inhibitory potential of the identified glycan residues. Of the glycans tested for their ability to prevent SSL5-platelet interactions, sLe^X^ was the most potent, followed by sLacNac. Sialic acid glycoside demonstrated a non-significant inhibition of SSL5. This was a surprising result as 16 of the 22 H-bonds possible between sLe^X^ and SSL5 are present on the much smaller sialic acid residue [22], highlighting the important role of the remaining six residues in the binding of sLe^X^ to SSL5.

Glycan-based therapeutic strategies are advancing towards clinical applications with sLe^X^ mimetics representing a promising new class of anti-inflammatory drugs [31], [32], [33], [34]. One such example, Bimosiamose, is currently in phase II trials in several disease applications [35], [36], [37]. We therefore chose to also test Bimosiamose in this scenario and found its inhibitory efficacy range to be 100 µM-1 mM, which is equivalent to the currently used human dose of 5–60 mg/kg.

In summary, we report the association of SSL5 with two platelet surface receptors, GPIbα and GPVI, which results in platelet activation and aggregation. We could identify several glycan structures as potential mediators of binding. Through this better understanding of the mechanisms involved in SSL5-platelet interactions, we describe novel inhibitory approaches, which are based on glycan structures. These data warrant further examination of SSL5 effects on platelets *in vivo*.

## Materials and Methods

A detailed description of the methods is provided in the expanded Methods section, available in the Supplemental Materials and Methods S1.

### Production of SSL5 and T175P (mutant SSL5)

The cDNA encoding SSL5 was isolated from *S. aureus* strain NCTC8325 and cloned into the pHOG21 vector for expression as a His-tagged fusion protein in *E. coli* BL21, then purified by Ni-affinity as described in Supplemental Materials and Methods S1.

### Glycan binding specificity by glycomics array

Based on previous reports on SSL proteins [14], [15], [22], glycans were mediate binding of SSL5 to glycoproteins. The printed mammalian glycan microarray at Core H of the Consortium for Functional Glycomics (Emory University School of Medicine, Atlanta) was used for screening of glycan-binding protein specificity, and providing a high-throughput screen for glycans that can bind to SSL5. Binding of SSL5 to 377 different glycans was evaluated, and 37 glycans were found to bind SSL5 according to stringent criteria detailed in Supplemental Materials and Methods S1.

### Statistical analysis

Unless otherwise noted, data are presented as Mean ± SEM. The statistical comparisons for the data were performed using ANOVA (following a Newman–Keuls test) in GraphPad Prism 5.0 Software, and differences were considered to be significant at *p*<0.05.

## Supporting Information

Materials and Methods S1 (DOC)
