# Supplementary material for: GPVI and GPIbα Mediate Staphylococcal Superantigen-Like Protein 5 (SSL5) Induced Platelet Activation and Direct toward Glycans as Potential Inhibitors
Source: PLoS One. 2011 Apr 28;6(4):e19190. doi: 10.1371/journal.pone.0019190 (PMC3084272; doi:10.1371/journal.pone.0019190)
Supplement: Materials and Methods S1 — (DOC) [file pone.0019190.s001.doc]

**Supplemental Materials and Methods S1**

**Production of SSL5 and T175P (mutant SSL5)**

*S. aureus* strain NCTC8325 was kindly provided by Dr. Howden (Microbiology Department, Monash University, Australia). The SSL5 gene, without the signal sequence [1], amplified by PCR (5’-GTT**CCATGG**CTAGTGAACATAAAGCAAAATAT­GAA-3’ ; 5’-GTT­**GCGGCCGC­­**TCT­AAT­GTT­GGC­TTCTATTTTTTC-3’, NcoI and NotI recognition sites under­lined, respectively) using *Vent_R_* DNA polymerase (Biolabs, New England) and subsequently cloned into the pHOG21 bacterial expression vector. T175P was constructed with a Quick-Change Site-Directed Mutagenesis Kit (Stratagene) according to the manufacturer’s instructions. Primers used were: sense primer 5’- TGAAAGATGGCG­GCTATTATCCGTTTGAA­CTTAA­TAA­AAA­ATT­ACA­AAC­­-3’ and antisense primer 5’- GTTTGTAATTTTTTATTAAGTTCAAACGGATAA­TAGCCGCCA­TCT­TTCA -3’. The resultant proline mutant sequence was verified to have the intended mutations, but not extraneous mutations, by ABI PRISM^TM^ 3100 Genetic Analyzer. After verification of the correct sequences of both SSL5 and T175P, the pHOG21/SSL5 and pHOG21/T175P expression vectors were transformed in BL21 *E. coli* as described previously [2,3]. Expression of His-tagged SSL5 and His-tagged T175P was induced with 1mM isopropyl-β-D-thiogalactopyranoside (IPTG; Sigma) for 4 hours at 37ºC. The His-tagged proteins were isolated in denaturing buffer (8M urea, 100mM NaH_2_PO_4_, 10mM Tris base, pH 8.0) and sonicated for 30 seconds (Branson Digital Sonifier; 40-50% Duty cycle; Output 4-5). His-tagged proteins were isolated using Ni-NTA Agarose (Invitrogen) affinity chromatography. Briefly were applied to a column containing 10ml activated Ni-NTA agarose equilibrated in denaturing buffer and the resin was washed twice with buffer containing 8M urea, 100mM NaH_2_PO_4_, 10mM Tris base, pH 6.3 and pH 5.9. Bound protein was eluted in 1.0ml fractions using an elution buffer (8M urea, 100mM NaH_2_PO_4_, 10mM Tris base, pH 4.5). Fractions containing eluted material were pooled and subjected to renaturation by dialysis against buffer containing 6M urea, 100mM NaH_2_PO_4_, 10mM Tris base, pH 7.4, for 2 hours, followed by 2 hours dialysis against the same buffer containing 4M urea. Finally, SSL5 and T175P were dialysed thoroughly overnight against phosphate-buffered saline (PBS) and stored in PBS at -80ºC before use. Protein purity was examined by sodium dodecyl sulfate polyacrylamide gel electrophoresis (SDS-PAGE) on a 12% slab gel using Comassie blue staining to visualise protein. Western blotting was performed using HRP-conjugated anti-His6 mAb and bound antibody was detected by enhanced chemiluminescence (ECL; Pierce Biotechnology).

**Human blood collection and platelet isolation**

The collection of human blood and the preparation of PRP or gel-filtered platelets (GFP) were undertaken as described previously [4]. GFP suspensions were adjusted to 8×10^6^ platelets/ml using modified Tyrode’s buffer (150mM NaCl, 2.5mM KCl, 12mM NaHCO_3_, 2mM MgCl_2_, 2mM CaCl_2_, 1mg/ml BSA, 1mg/ml dextrose; pH 7.4) for analysis by flow cytometry, or to 2.4×10^7^ platelets/ml using modified Tyrode’s buffer for static adhesion experiments. Washed platelets were prepared as previously described [5]. Briefly, PRP was acidified to pH 6.5 using acid citrate dextrose (ACD) buffer (38mM citric acid anhydrous, 75mM sodium citrate, 136mM D-glucose, pH 5.0) and treated with 1μM prostaglandin E1 (PGE1, Calbiochem, CA, USA). PRP was then centrifuged at 800×*g* for 15 minutes and the platelet pellet was washed twice in buffer containing 140mM NaCl, 5mM KCl, 12mM Na citrate, 10mM glucose and 12.5mM saccharose pH 6.5, followed by centrifugation at 800×*g* for 10 minutes. Washed platelet pellets were resuspended in Tyrode’s buffer at platelet concentrations appropriate for each experiment.

**Activation of platelets by SSL5**

Platelet P-selectin and activated GPIIb/IIIa levels were used to estimate platelet activation triggered by treatment with SSL5. Samples of PRP diluted 50-fold in PBS buffer, were incubated with either vehicle (PBS), 30μg/ml SSL5 mutant T175P, 20µM ADP (Sigma), 100ng/ml convulxin (Pentapharm, Switzerland) or 30μg/ml SSL5 at 37°C for 15 minutes. Saturating amounts of mouse anti-human P-selectin-PE and PAC-1-FITC (BD Biosciences, San Jose, California) were added to human PRP samples. Isotype control antibodies were used in each experiment.

**Aggregation of platelets induced by SSL5**

Washed platelets were prepared as described above. Light transmission aggregometry was performed at 37°C, with constant stirring in a Helena AggRAM™ aggregometer (Helena, USA) and induced by addition of indicated concentrations of SSL5 diluted in PBS. Aggregations were recorded for 10 minutes.

**Analysis of SSL5 binding to platelet receptors using immunoprecipitation**

Washed platelets (3x10^8^ platelets/ml) were lysed in ice-cold lysis buffer containing 50mM Tris-HCl (pH 7.4), 150mM NaCl, 1% Triton X-100, Protease inhibitor cocktail (Roche, Basel, Switzerland), 10mM NaF, 1mM Na_3_VO_4_ and 10mM NaP_2_O_7_ and sonicated for 10 seconds. Sonicated samples were then clarified by centri­fugation at 1000x*g* for 10 minutes at 4°C. Platelet lysates were pre-cleared by incubation with 30μl of protein G-sepharose resin (Amersham) for 1 hour at 4ºC. The sepharose beads were pelleted, and equal volumes of the cleared supernatant were transferred to fresh tubes containing recombinant SSL5 protein (5μg) and 1μg of either anti-GPIbα WM23 or mouse IgG1. After incubation for 2 hours at 4ºC, 30μl of protein G-sepharose resin was added to each tube and incubated overnight at 4ºC to capture immune complexes. Resins were pelleted and washed with lysis buffer 5 times and finally eluted in 40μl of SDS-sample loading buffer and heated to 95ºC for 10 minutes. Equal volumes of sample were separated on a 12% SDS-PAGE gel and electrotransferred onto Hybond-C nitrocellulose membranes (Amersham). Immunoblots were probed with anti-GPIbα mAb WM23 (1:1000) and anti-His·Tag (1:3000) (Novagen, Madison, WI) followed by HRP-conjugated secondary antibodies. Bound antibodies were detected using ECL.

**SSL5 binding to HL60 cells and platelets**

HL60 cells (ATCC: CCL-240) were maintained in RPMI 1640 medium containing 10% fetal bovine serum supplemented with 2mM L-glutamine, penicillin (100IU/ml) and streptomycin (100μg/ml). HL60 cells (5×10^6^ cells/ml) or GFP (8×10^6^ platelets/ml) in modified Tyrode´s buffer were incubated with increasing concentrations of SSL5 in the presence of 0.5mg/ml human serum albumin (Sigma) for 30 minutes on ice. Samples were pelleted by centrifugation and resuspended twice, then Alexa Fluor 488-conjugated anti-Penta·His (Qiagen, Hilden, Germany) was added and cell suspensions were incubated on ice for 20 minutes in the dark. Samples were then fixed by addition of CellFIX (BD Biosciences) and surface-associated fluorescence was measured by flow cytometry (FACSCalibur, BD Biosciences) as described in Bassler *et al* [4].

**Analysis** **of platelet-SSL5 interactions using mAbs against platelet receptors**

The interaction between SSL5 and GPIbα proteins on platelets was examined by first incubating washed platelets (8×10^6^ platelets/ml) with 3μg/ml of anti-GPIbα mAbs SZ2 (BD Biosciences, San Jose, California), AK2, BX1 or WM23 for 15 minutes at 37°C. 0.3-30μg/ml SSL5, or mutant SSL5 (T175P) as a negative control, were then added and incubated for 15 minutes at 37°C. Subsequently, Alexa Fluor 488-conjugated anti-penta-His was added and incubated for 15 minutes at 37°C. Samples were fixed using 1xCellFix and analysed by flow cytometry in a FACScalibur. Samples were also tested using iso-FITC and goat anti-mouse IgG-FITC to control for non-specific binding, and non-displacement of anti-GPIbα mAbs, respectively.

Recombinant GPVI ectodomain was assessed for its ability to inhibit SSL5-induced platelet activation *in vitro* by measuring P-selectin levels by flow cytometry. GPVI ectodomain (0.74μM) was preincubated with SSL5 (10μg/ml, 0.37μM) for 15 minutes at 37°C then mixed with PRP for 15 minutes at 37°C. Anti-P-selectin antibody was then added for 15 minutes then the sample was treated with CellFIX and analysed by flow cytometry. Levels of P-selectin were compared with levels in PRP samples that were untreated or had been treated with 20μM ADP, or 10μg/ml SSL5 or T175P SSL5. These experiments were undertaken using six healthy donors.

**Interaction between SSL5 and platelet receptors GPIbα and GPVI using surface plasmon resonance technology**

Surface plasmon resonance binding studies to detect interactions between proteins were conducted using a Biacore T100 machine (BIAcore AB, Uppsala, Sweden). Recombinant, Fc fusion proteins of GPVI (encompassing amino acids 21-234 of GPVI) and GPIbα-(amino acids 1-290) were expressed in mammalian cells, and affinity-purified and concentrated as previously described [6]. Purified GPVI-Fc and GPIbα-Fc were biotinylated using NHS biochemistry and dialysed extensively against 10mM Hepes (pH 7.4), 150mM NaCl and 0.005% polysorbate 20 surfactant (HBS buffer) then concentrated using an Amicon filtration device (Millipore). All experiments were performed in HBS buffer. The biotinylated proteins were then immobilized at the required levels (500–700 RU) by injection over CM5-streptavidin biosensor chips according to manufacturer’s instructions. Non-specific interactions were controlled for by subtraction of the signal from a reference flow cell coated with an irrelevant biotinylated Fc-containing protein. The SSL5 protein used in binding studies was monomeric. To avoid avidity effects with GPVI and GPIbα, which were expressed as dimeric Fc-fusion proteins, experiments were only performed with either GPVI or GPIbα immobilised on the chip surface. Binding data was evaluated using Biacore T100 Evaluation software (BIAcore AB, Uppsala, Sweden).

**Adhesion of platelets to fibrinogen under static conditions**

Glass cover slips (12mm circular, No 1; Fisher Scientific) were coated with 30μg/ml fibrinogen (Sigma) at 4°C overnight. After washing with modified Tyrode’s buffer, the cover slips were blocked with 1% BSA (bovine serum albumin, Sigma) and washed twice with modified Tyrode’s buffer. GFP (2.4×10^7^ platelets/ml) were incubated for 30 minutes at 37°C with 1, 5 and 10μg/ml SSL5, using PBS or 10μg/ml T175P as a negative control and 20μM ADP as a positive control. The cover slips were then washed twice with modified Tyrode’s buffer, fixed with CellFIX for 15 minutes and mounted with Vectashield mounting medium (Vector laboratories, Burlingame, CA). The DIC (×60) was assessed using an F-View II digital camera.

**Effects of neuraminidase on SSL5 binding to platelets**

GFP (2×10^6^ platelets/ml) in G1 buffer were incubated in the presence or absence of (final concentration) 100IU/ml neuraminidase (New England Biolabs) for 60 minutes at 37°C and washed once with Tyrode’s buffer. Pellets were resuspended in modified Tyrode’s buffer at 8×10^6^ platelets/ml, then 1μg/ml SSL5 was added and incubated for 15 minutes at 37°C. Following this, Alexa Fluor 488-conjugated anti-penta·His mAb was added and incubated at room temperature for 20 minutes in the dark. Finally, CellFIX was added and antibody binding was assessed by flow cytometry.

**Determination of glycan binding specificity by glycomics array**

A sample of purified SSL5 was sent to Core H of the Centre for Functional Glycomics (http://www.functionalglycomics.org), located at Wayne Rollins Research Centre, Atlanta, GA. The printed mammalian glycan microarray format at Core H of the Consortium for Functional Glycomics (Emory University School of Medicine, Atlanta) has replaced the earlier ELISA-based microplate array for routine screening of glycan-binding protein specificity, and provides a high-throughput screen for glycans that can bind to SSL5. The sample, at a concentration of 200μg/ml, was labelled with anti-penta-His fluorescent antibody and incubated with an array of 377 glycan structures as listed on (http://www.functionalglycomics.org/static/consortium/­resources/resourcecoreh8.shtml) then covalently linked to NHS-activated glass microscope slides and analysed for fluorescence activity. The relative binding was expressed as relative fluorescence units (RFU). An SSL5-binding glycan was defined as any glycan, which bound to a level exceeding two standard deviations above the mean of all values below the background level (10% of the strongest binding glycan).

**Effect of glycans on SSL5-induced platelet activation *in vitro***

The glycans including sialyl Lewis X antigen (sLe^X^; from Carbosynth Limited, Berkshire, UK), sLacNac (Neu5Acα2-3Galβ1-4-GlcNAc; Dextra Laboratories, Earley Gate, UK), Bimosiamose (Anthem Biosciences, Bangalore, India) and sialic acid glycoside (Carbosynth Limited, Berkshire, UK) were screened for their ability to inhibit SSL5-induced P-selectin expression on human washed platelets by flow cytometry. Vehicle alone, or 100μM of either sLe^X^, sLacNac or sialic acid glycoside glycans, or Bimosiamose (10μM – 1mM) were preincubated with 10μg/ml SSL5 or 20μM ADP for 15 minutes at 37°C then incubated with PRP diluted 1:50 in Tyrode’s buffer for 15 minutes at 37°C. Saturating amounts of isotype control or anti-P-selectin antibody were then added to the platelet suspensions, incubated a further 15 minutes then all samples were treated with CellFIX and analyzed by flow cytometry. P-selectin levels were also assessed in untreated samples and samples treated with 10μg/ml T175P, or each of the putative SSL5-inhibitors alone and compared with P-selectin levels in PRP samples that had been treated with 20μM ADP.

References

1. Baba T, Takeuchi F, Kuroda M, Yuzawa H, Aoki K, et al. (2002) Genome and virulence determinants of high virulence community-acquired MRSA. Lancet 359: 1819-1827.

2. Hagemeyer CE, von Zur Muhlen C, von Elverfeldt D, Peter K (2009) Single-chain antibodies as diagnostic tools and therapeutic agents. Thromb Haemost 101: 1012-1019.

3. Schwarz M, Meade G, Stoll P, Ylanne J, Bassler N, et al. (2006) Conformation-specific blockade of the integrin GPIIb/IIIa: a novel antiplatelet strategy that selectively targets activated platelets. Circ Res 99: 25-33.

4. Bassler N, Loeffler C, Mangin P, Yuan Y, Schwarz M, et al. (2007) A mechanistic model for paradoxical platelet activation by ligand-mimetic alphaIIb beta3 (GPIIb/IIIa) antagonists. Arterioscler Thromb Vasc Biol 27: e9-15.

5. Patel YM, Patel K, Rahman S, Smith MP, Spooner G, et al. (2003) Evidence for a role for Galphai1 in mediating weak agonist-induced platelet aggregation in human platelets: reduced Galphai1 expression and defective Gi signaling in the platelets of a patient with a chronic bleeding disorder. Blood 101: 4828-4835.

6. Gruner S, Prostredna M, Koch M, Miura Y, Schulte V, et al. (2005) Relative antithrombotic effect of soluble GPVI dimer compared with anti-GPVI antibodies in mice. Blood 105: 1492-1499.
